# Postoperative treatment after partial nail ablation of ingrown toenails — does it matter what we recommend? A blinded randomised study

**DOI:** 10.1080/02813432.2019.1608041

**Published:** 2019-05-03

**Authors:** S. V. Bernardshaw, Liv Helene Dolva Sagedal, Kristin Møystad Michelet, Christina Brudvik

**Affiliations:** a Acute and Emergency Department, Haukeland University Hospital, Bergen, Norway;; b Elverum Hospital, Elverum, Norway;; c Ektorp Medical Centre, Stockholm, Sweden;; d Department of Clinical Medicine, University of Bergen, Bergen, Norway

**Keywords:** ingrown toenail, infection, pain, soapbath, bandage, postoperative treatment

## Abstract

**Trial design:** In this blinded randomized study we analyzed patient reported outcome of three different treatments after nail surgery. We compared daily footbath with either alkaline or acidic soap or just a simple bandage of gauze dressing.

**Method:** After partial nail ablation surgery, patients were randomized into three postoperative treatment modalities. Outcome in terms of reduction in pain, improvement of function, reduction of signs of infection and postoperative soothing effect were reported after one and two weeks. A generalized linear mixed model was used to analyze possible statistical differences between the groups.

**Results:** 97 patients, 57% women, mean age 31 years, were included. Men reported significantly less pain and better function than women. Despite a registered lower growth of invasive pathogenic microbes following the use of acidic soaps, this did not lead to less infections than in the groups using either alkaline soap baths or bandaging. On the contrary, patients keeping the bandage on had significantly lower signs of infection after one week. Two patients using soap baths had growth of MRSA. Two weeks postoperatively, all three treatment alternatives had similar patient reported outcome in all parameters, and nobody needed antibiotics.

**Conclusions:** This prospective randomized study was unable to prove that footbath with either acidic or alkaline soap should be preferred to just leave the postoperative bandage on for a week after partial nail ablation. We recommend that postoperative advice should be given on an individual basis, especially since our study did not involve patients with high risk of infections.

## Introduction

Onychocryptosis or ingrown toenail (IGTN) is a common condition in adults and children, and often causes pain, difficulty walking and psychosociological distress. Without proper care or treatment, it may progress into a secondary infection, paronychia, of varying severity [1,2]. Numerous conservative and surgical methods of IGTN treatment exist. Partial ablation of the nail and nail matrix, with or without chemical cauterisation, is becoming the surgical method of choice [2].

Despite the availability of many postoperative treatment options following nail ablation, none have been assessed in formal studies. We aimed to study the patient-reported effect of three different treatments after nail surgery: footbath with alkaline soap, footbath with acidic soap and simple gauze dressing. Daily footbaths with two different soaps are traditionally recommended in Norway both for the prevention of IGTN and postoperatively after partial nail ablation. The soaps that are used are Pine-sol (“grønnsåpe”, pH 10) and Lactacyd (pH 4). Topical or systemic antibiotics are not used unless the patient is immunocompromised or presents with comorbidities such as diabetes [2].

This study aimed to evaluate the patient-reported parameters of pain, toe function, indicators of infection and the general soothing effect of the different treatment options.

## Materials and methods

### Design

The present blinded, block-randomised study involved 97 patients with an IGTN who were reported to have attended Bergen Accident and Emergency Department (A&E) during the seven months from November 2014 to May 2015. After surgery, patients were randomised into three different postoperative treatment groups. Outcomes were recorded after one and two weeks.

In Norway, IGTN is typically treated by general physicians (GP) with some experience in minor surgery. In the 97 patients who presented to Bergen A&E and were included in this study, the affected nail plate was surgically removed by partial nail ablation (PNA).

Recurrences of IGTN often require complete nail ablation using phenol (chemocautery). The A&E doctors were mostly GPs or doctors with specialisations working on rotation, but all were trained to perform routine PNA and chemocautery. The medical staff were informed about the study, both by lectures and by written information ahead of the study start.

### Participants

**Figure 1.**
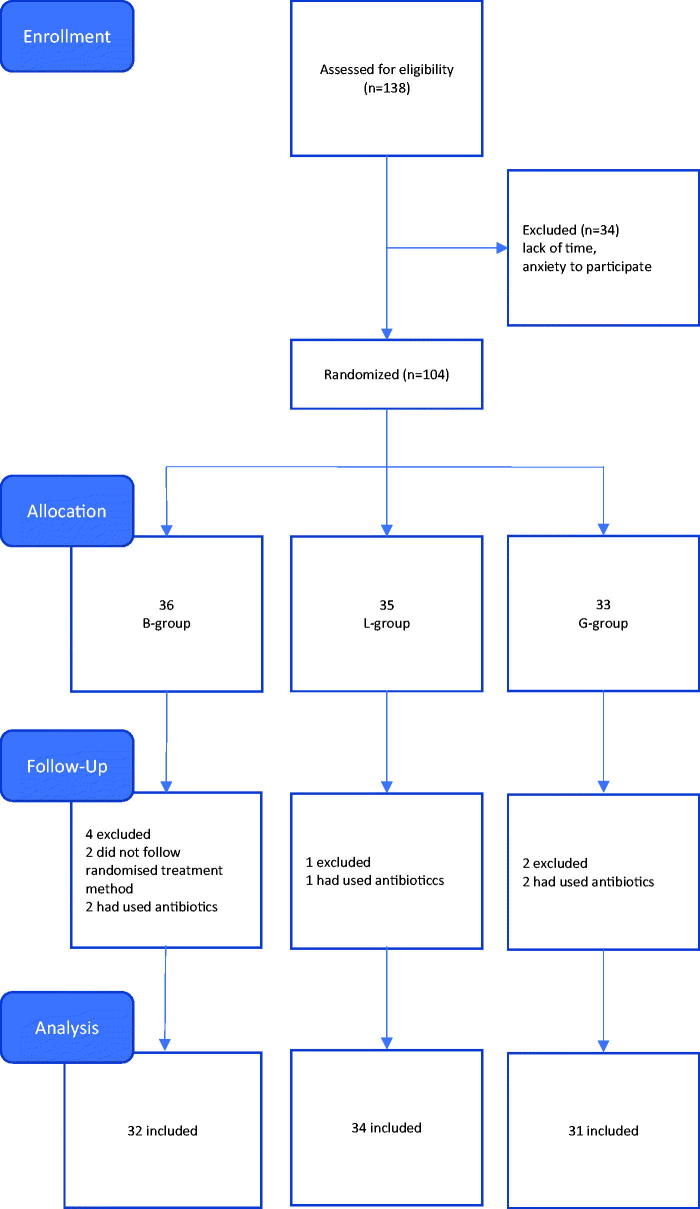
Flow chart of the block-randomized controlled trial of partial nail ablation.

Patients aged 16 years and older who received surgical treatment for IGTN at Bergen A&E during the seven months period from Nov 2014 to June 2015 were included in the study. We excluded patients who had an increased risk of acquiring infections (due to immunocompromising conditions or treatments, pregnancy, diabetes or peripheral vascular disease) as well as patients who had used antibiotics during the past two weeks. Initially, 138 patients were enrolled in the study, but 34 were excluded due to either lack of time or anxiety to participate. (Figure 1).

### Sample size

The sample sizes were decided based on differences in signs of infection between treatment groups. Previous studies of toenail excisions [3] and wound sutures in an A&E department [4] have reported that 20% and 15% (respectively) had some degree of postoperative infection.

Based on this we assumed that a difference of 10% between the two treatments could be considered clinically significant. To detect the difference between the treatments with a power of 80% (*α* = 0.05) a total of 30 patients were required per study group. A difference of two or more patients would then be significant.

### Ethics

Signed informed consent was obtained from the patient and the family/guardian of patients aged 16 and 17 years. The study was approved by the regional committee for ethics (approval number: 2014/1502/REK sør-øst).

### Stages of ingrown toenail

Patients’ clinical conditions were registered by the physician and the severity of infection and inflammation were graded according to commonly accepted stages [2,5]:

Stage 0: Normal toenail and lateral nail fold
Stage 1: Local irritation with erythema, oedema and pain at the lateral nail fold (paronychia), without gross infection or granulation tissue.
Stage 2: Infection of the nail border with pus and hypertrophic granulation tissue, with or without a history of onychocryptosis.
Stage 3: Infection of the nail border with hypertrophic granulation tissue on the lateral nail fold and a previous history of onychocryphosis.

### Surgical treatment procedure

The affected side of the nail plate was surgically removed by PNA. Recurrences of IGTN were treated with PNA coupled with chemocautery using 90% phenol applied to the nail matrix for four minutes to prevent regrowth. However, chemocautery was not performed in patients with stage 2 IGTN until the infection had declined following preliminary treatment by PNA. The procedures were performed using a peripheral nerve block of 1% lidocaine hydrochloride without adrenaline at the base of the toe. The operating site was covered with paraffin gauze (Jelonet) under a dry bandage following surgery. Neither topical nor systemic antibiotics were used.

### Postoperative treatment groups

Three postoperative treatment groups were established:

Group B — No treatment, the toenail remained covered with the original paraffin gauze (Jelonet) until one week after surgery.
Group G — a non-antimicrobial detergent-based product (Pine-sol soap) containing esterified fatty acids and potassium hydroxide (*pH* = 10) was used for daily footbaths. The soap was non-perfumed, liquefied and commercially available in drugstores and pharmacies. The G group patients were instructed to soak the toe in one tablespoon of Pine-sol soap diluted in 1 litre of lukewarm water daily for 15 minutes. The area was washed with fresh water and dried before applying a new dressing.
Group L — Lactacyd is a lactoserum-lactic acid compound which changes the pH environment of the skin (*pH* = 4). It is commercially available in pharmacies in a liquefied form. The L group patients were instructed to use 2 tablespoons of Lactacyd soap diluted in 1 litre of lukewarm water daily for 15 minutes. The area was washed with fresh water and dried before applying a new dressing.

If patients experienced increasing pain or other signs of infection, they were advised to contact Bergen A&E ahead of the planned assessment at 5–7 days (hereafter referred as one week) postoperatively.

### Outcome parameters

We focused on four main outcome parameters: pain, function of toe, infection and the patient's perception of the soothing effect of postoperative treatments.

Pain was reported by the patient using a visual analogue scale (VAS) of a straight line from 0 to 10 with the endpoints defining extreme limits as “no pain at all” and “pain as bad as it could be”. At follow-up interviews, the level of pain was reported by telephone using a similar numeric rating scale (NRS).

Function of the toe was reported by the patient as the reduced ability to move the first toe, defining no reduction in function as 0 and total immobility as 10 by VAS. At follow-up interviews, reduced function was assessed by NRS by telephone.

Infection was initially graded by the physician then by the patients as “yes” or “no” for redness, swelling and oedema, during the telephone interviews. Bacteriological samples using the standard kit, from the nail bed wound were analysed one week after surgery.

The patients’ perception of the soothing effect of postoperative treatment was categorised into three alternatives; either “yes” (soothing), “no” (not soothing) or “uncertain”.

### Data registration, blinding, randomisation and collection

At the first attendance, both the patient and attending physician filled out a questionnaire. The patient was asked about symptoms, previous treatments and problems related to IGTN. The physician assessed the stage of infection. After surgery, the patients were randomly allocated into one of the three postoperative treatment groups, using pre-arranged sealed and mixed envelopes designed by the co-authors (KM and LS). In order to achieve a uniform distribution in a limited number of patients, we placed blocks of 30 envelopes at a time to be randomly selected, containing ten of each of the three treatment alternatives. The staff and authors were blinded to the assignment of treatment and the envelopes were chosen by patients. Some patients (*n* = 3) with bilateral involvement underwent the same procedure for both sides. The patients were examined one week after the procedure by staff nurses. Symptoms of possible infection were noted, and bacteriological samples were taken. The patients themselves reported on predefined outcome parameters and were specifically asked whether they had followed the recommended instructions or used any antibiotics. Two patients in the bandage group were excluded because they chose to use footbaths with soap. Five patients were excluded because they had used either local antibiotic cream on the toe or systemic antibiotics for other reasons than the toe nail infection (Figure 1). Complete recovery was expected within three weeks. After two weeks, the remaining 97 patients were contacted by telephone and reported the outcome parameters once more. The interviews were performed by the co-authors (KM and LS).

### Data management and statistics

Descriptive statistics for the study population were derived from the mean values and standard deviation (SD) for continuous variables. Data for different registration periods — baseline (T0), one week postoperative (T1) and two weeks postoperative (T2) — were analysed by random intercept, mixed logistic regression models. Adjustments for gender were performed for the analysed parameters at baseline. Gender is a confounder in the assessment of pain [6], and pain influences both toe function and the perception of the soothing effect. An alpha level of 0.05 was used for all statistical tests. The Statistical Package for the Social Sciences version 17 (SPSS, Chicago, IL, USA) was used for statistical analysis.

## Results

From November 2014 to May 2015 a total of 97 patients with ingrown toenail were assessed, 57% were women and the mean age was 31 years (Table 1).

**Table 1.** Characteristics of the study participants. Baseline data:

Variable	Level	B group (*n* = 32)	L group (*n* = 34)	G group (*n* = 31)	Total (*n* = 97)
Age	mean (SD)	29.9 (18.0)	34.2 (20.0)	29.8 (14.2)	31.4 (17.6)
Gender	men	17 (53.1)	14 (41.2)	11 (35.5)	42 (43.3)
	women	15 (46.9)	20 (58.8)	20 (64.5)	55 (56.7)
Pain	mean (SD)	5.0 (2.6)	5.9 (2.3)	6.0 (2.4)	5.6 (2.4)
Function	mean (SD)	3.4 (2.6)	4.2 (2.9)	5.1 (2.6)	4.2 (2.8)
Infection	0	2 (6.7)	4 (12.1)	5 (16.1)	11 (11.7)
Grades (%)	1	12 (40.0)	7 (21.2)	5 (16.1)	24 (25.5)
	2	9 (30.0)	12 (36.4)	13 (41.9)	34 (36.2)
	3	7 (23.3)	10 (30.3)	8 (25.8)	25 (26.6)

Participants were divided into three groups, the acidic Lactacyd soap (L group), alkaline Pine-sol soap (G group) and the Jelonet-gauze bandage (B group).

### Background data of the population

Of the patients included in this study, 86% reported that they had tried treatment of IGTN by footbaths with soap prior to their admission to A&E. However, the soap treatment failed to prevent the need for toenail surgery. Seven patients (7%) were treated bilaterally, and one patient was treated twice during the study period, hence, 104 procedures were performed.

Chemocautery with phenol was performed in 18% of the patients (*n* = 17).

Most of the patients (70%) had experienced IGTN before 25 years of age and were operated on before the age of 25. Of the study population, 43% were teenagers. Twenty one percent of the patients were operated on for the first time, 21% for the second time and the rest had been treated more than twice. Three patients (3%) received treatment for both (left and right) toes at the same time and seven patients were treated for infections in the nail borders on both sides of the same toe. Left sided and right sided toes represented 49% and 47% respectively. Major predisposing factors were familial disposition (52%) and use of constrictive footwear (75%).

### Baseline characteristics after randomisation

**Figure 2.**
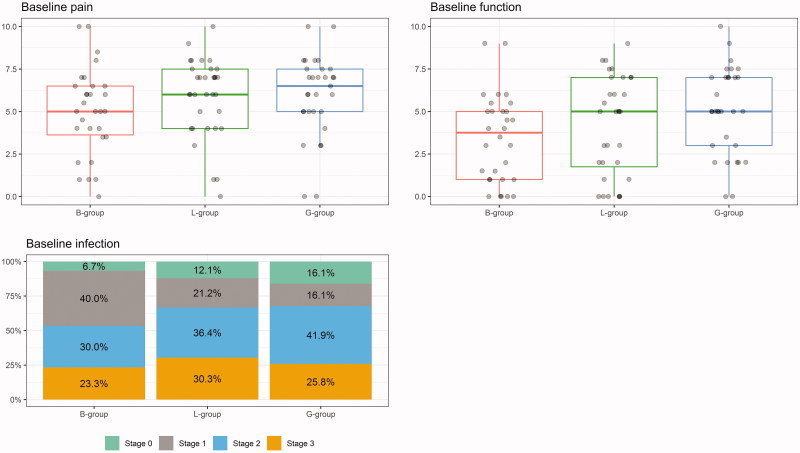
(a–c) Box plots illustrating the median (bold horizontal line) and inter quartile range (square box) of Visual Analogue Scale (VAS) from 0 to 10 of respective pain scores (a) and function scores (b) before treatment (baseline). The columns with colours (c) illustrate the distribution of stages of infection (0–3) in the ingrown toenails before treatment in the different postoperative treatment groups; bandage (B-group, *n* = 31), acidic soap (L-group, *n* = 34) and alkaline soap (G-group, *n* = 32).

Despite the blinded randomisation, male patients represented a greater proportion of the bandage group than either soap treatment groups and reported less pain and better function at baseline (Table 1), (Figure 2). After stratifying by gender, we found that men in general reported less pain and better function than women. Thus, in our analysis of outcome after one and two weeks, we adjusted for both gender and baseline pain and function. Painkillers such as paracetamol/NSAIDS were used by 35% of patients during the study period. The use of painkillers or antibiotics during the observational period did not influence the outcome (data not shown).

### Pain

**Figure 3.**
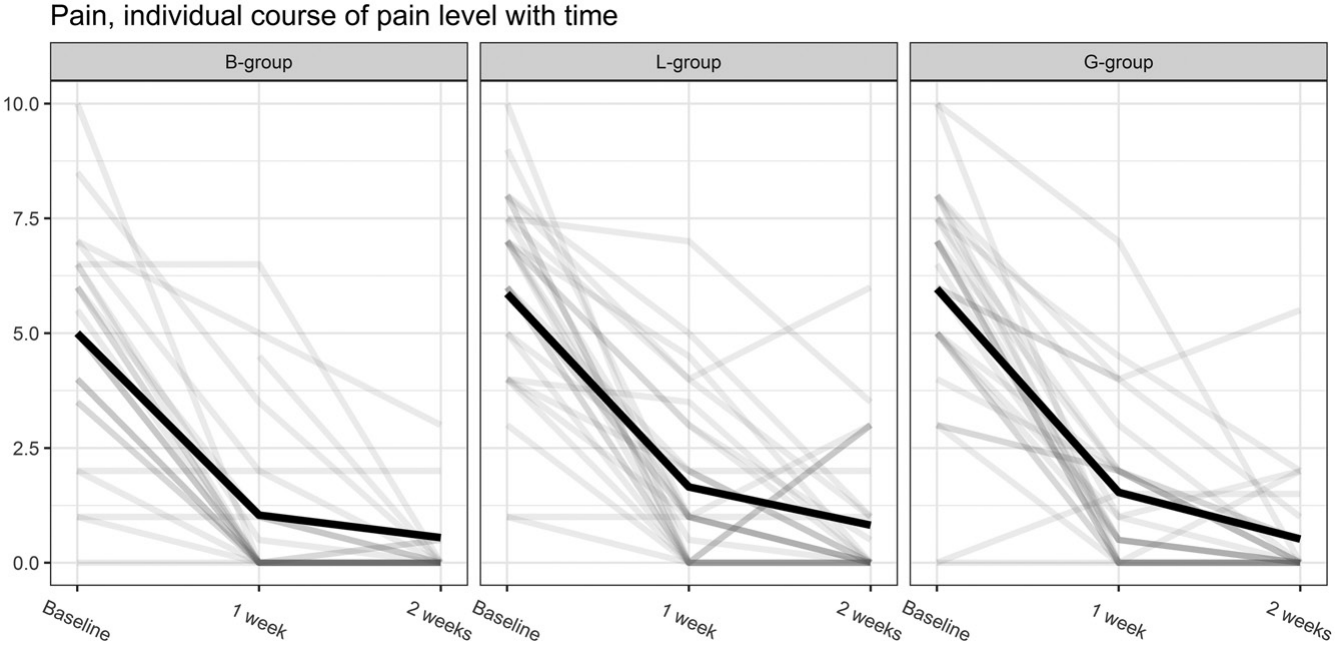
Changes in pain level as the patients through Visual Analogue Scale (VAS) (after one week) and Numerical Rating Scale (NRS) reported a response to the three different treatments (after two weeks). B: Bandage, L: Lactacyd soap, G: Pine-sol soap.

We used a generalized linear mixed model, and after adjustments for gender as a confounder we found that the reduction of pain after one week were significantly higher in the alkaline soap group compared to the bandage group (Figure 3). Still, patients in both soap groups were more likely to have pain compared to patients in the bandage group. However, after two weeks the differences in the reduction of pain between the groups were no longer present (Figure 3). In general, patients who had received chemocautery reported more pain after one week than other patients.

### Function

The unadjusted risk of reduced function after one week was 30% lower (*OR* = 0.7) in the acidic soap group and 40% lower (*OR* = 0.6) in the alkaline soap group compared with the bandage group. After adjusting for gender as a confounder, these differences were still present, but not significant due to wide confidence intervals (CI). After two weeks the differences between the groups had diminished further (Table 2).

**Table 2.** Risk analysis of outcome parameters under various conditions after one week (T1) and two weeks (T2).

		T1				T2			
		OR	Upper	Lower	p	OR	Upper	Lower	p
Pain	L *vs* B	3.9	0.1	15.1	0.05	2.4	0.6	9.5	0.2
	G *vs* B	4.5	1.1	18.4	0.04	1.7	0.4	7.3	0.5
Function	L *vs* B	0.7	0.2	2.8	0.6	0.9	0.1	5.5	0.9
	G *vs* B	0.6	0.1	2.6	0.5	1.1	0.2	7.3	0.9
Infection	L *vs* B	6.7	1.8	24.9	0.005	0.6	0.2	2.2	0.5
	G *vs* B	6.0	1.6	23.3	0.009	1.5	0.4	5.3	0.5

OR: odds-ratio, T1: one week after surgery, T2: two weeks after surgery, L: Lactacyd soap group, B: Bandage group, G: Pine-sol group.

### Infection

The distribution of infection at baseline is shown in Figure 2c. In comparison with the bandage group, the unadjusted risk of signs of infection after one week was 6.7 times higher in the acidic soap group (*OR* = 6.7) and 6 times higher in the alkaline soap group (*OR* = 6.0). Following adjustment for gender, the difference to the bandage group was still significant; *p* = 0.005 for the acidic soap and *p* = .009 for the alkaline soap groups (Table 2). However, after two weeks there were no significant differences. In general, patients who had received chemocautery had fewer signs of infection at baseline than other patients.

### Soothing effect

The perception of the soothing effects of postoperative treatments were categorised as positive in most patients (55%), while 20% found it negative and 25% were uncertain. Fewer patients in the bandage group reported that the treatment was soothing, but the difference between the bandage and either soap group was not significant (*p* = 0.177). In general, patients who had received chemocautery reported a lower soothing effect after one week than other patients.

### Bacterial growth

Microbiological samples of the nail bed were available from 79% of the patients included in the study. From the 95 cultures, *Staphylococcus aureus* was the predominant bacteria (58%) and 43% of all *Staphylococcus* genera showed beta lactamase resistance, including MRSA (2%). *Staphylococcus aureus* growth was present in 46% of the acid soap group, 71% of the alkaline soap group and 56% in the bandage group (Table 3). Some cultures showed growth of a mixture of microorganisms which represented normal wound colonisation. Two patients had growth of MRSA. We found no significant difference in the growth of pathogenic bacteria between the different treatment groups, nor did the infection reported by the patients match the bacteriological results.

**Table 3.** Percentage of different types of bacteria identified in infected toe nails in the randomized treatment groups with different stages of paronychial infection.

Group	Microorganisms	Percentage of infected toe nails			
		Grade 0	Grade 1	Grade 2	Grade 3
Bandage (B)	*Staphylococci*	–	23%	30%	3%
	Mixed	–	3%	3%	–
Lactacyd (L)	*Staphylococci*	–	30%	18%	–
	Mixed	–	3%	6%	–
	MRSA		3%	–	–
Pine-sol (G)	*Staphylococci*	3%	32%	32%	–
	Gram neg Rods	–	3%	3%	
	Strepto/Coryne	–	–	3%	–
	MRSA	–	–	3%	–

The following *Staphylococci* were identified: *S. aureus, S. epidermis and S. lugdenisis.*

Key: Mixed: see text, MRSA: Methicillin-resistant *Staphylococcus aureus,* Strepto: Beta-hemolytic *Streptococcus*, Coryne: *Corynebacterium.*

## Discussion

Our study is the first to compare three postoperative treatments of IGTN that are available in day-to-day practice. We carried out a patient-oriented analysis of the effect of these

treatments. The main conclusion of our findings is that the three treatment alternatives have similar effects after two weeks in terms of pain reduction, improvement of function, reduction of signs of infection and postoperative soothing effect. It was an unexpected result that keeping the original paraffin gauze (Jelonet) on until one week after surgery would show significantly lower signs of infection than the soap bath treatments. However, this significant difference vanished by two weeks post- surgery, and the treatment alternatives can be regarded as equal.

The subungual area of the nail often harbours high numbers of microorganisms which are also found in the adjacent skin area. Artificial nails and chipped nail polish are associated with a further increase in the number of bacteria on fingernails [7–9]. The microbial flora of the skin consists of resident (colonising) and transient (contaminating) microorganisms. Resident microorganisms such as the coagulase-negative *staphylococci*, members of the *Corynebacterium* genus (diphtheroids or coryneforms), *Acinetobacter* species and occasionally members of the *Enterobacteriaceae* group cause infections after invasive procedures. In our study we found two patients with MRSA. We consider these patients as insidious cases, since they had neither been travelling abroad in high risk areas nor been admitted to hospitals. This surprising finding required extended contamination control at our surgical section and additional infection follow-up of the patients by their GPs. Norway has previously had a low prevalence of MRSA-infections, but this has increased in recent years, even outside hospitals [10].

Although the growth of microorganisms was lower in the patients treated with acidic soap than in the other groups, the difference was not significant and did not correspond with clinical signs of infection. In a previous study it was found that washing with low pH soap influenced not only the pH on the skin surface in the short term, but also the cutaneous microflora in the long term [11]. The acid mantle of the skin plays an integral role in the function of the skin barrier as well as regulating the bacterial flora [11]. Other researchers have highlighted the negative effects of common cleaning agents and have even argued that normal tap water may have a negative effect on the skin surface [12]. Increasing the skin pH irritates the physiological protection of the acid mantle, changes the composition of the cutaneous bacterial flora and affects the activities of enzymes in the upper epidermis. Soaps with a pH of about 5.5, such as Lactacyd, appear to mimic the normal skin environment well, and, in contrast to alkaline soap, do not interfere with the cutaneous microflora [13]. The skin pH normally ranges from pH 4 to 6, and the normal bacterial flora is optimal at these slightly acidic pH levels, whereas pathogenic flora such as S. *aureus* thrive at neutral pH levels [14]. In this study, we found lower pathogenic growth in the Lactacyd group than in the other treatment groups (Table 3). However, our study was unable to confirm that the lower skin pH following acidic soap baths resulted in reduced clinical signs of infection.

The alkaline properties of Pine-sol soap promote dissolving and destabilisation of the skin barrier. This can, in theory, cause enhanced debridement and discharge of necrotic tissue. Despite minor indications of improved toe function during alkaline soap bath treatment, this improvement was not significant compared to the other groups. However, in our study, both soap baths seemed to result in temporary improvement in toe function, contrary to the toe in gauze (B group) that was immobilized during the first postoperative week.

In general, patients who underwent chemocautery had lower signs of infection at baseline compared with the other patients due to the exclusion of patients with stage 2 and 3 infections. The risk of postoperative complications is higher in patients with ongoing aggressive toe infections. Those who underwent chemocautery reported less postoperative pain relief after one week than other patients. This is likely due to chemical inflammation.

As anticipated from previous studies [6], men generally reported less pain than women in all groups.

The overall healing time for all three groups was approximately two weeks regardless the mode of postoperative treatment. None of our patients developed postoperative infections which required antibiotics. Although it is likely that eliminating the cause of the IGTN infection with PNA surgery will be sufficient to prevent secondary infections, patients with increased risk of infection were excluded from this study as they require shorter intervals for medical wound control than one week. Concomitant use of antibiotics for IGTN is not our A&E department’s or national policy, except for those at high risk of infection. The fact that all our patients recovered during the observation period confirms the validity of our restrictive approach to the use of antibiotics.

## Limitations

Despite the block-randomised and blinded design of the study, patient numbers were not sufficient to be able to provide robust, evidence-based recommendations for postoperative care of IGTN.

It has been shown that the use of phenol was associated with an increased rate of postoperative infection compared with not using phenol [15]. In our study, 17% of the participants received phenol cauterisation, and it was considered that their inclusion may have influenced our analysis. Fortunately, these patients were evenly distributed in the three treatment groups and did not affect the findings.

Most patients in our study had been treated more than once for the same condition and had experienced similar pain before. It is known that the experience of pain may change in chronic conditions compared to acute conditions, but the distribution of patients with previous experience of IGTN were similar between groups.

## Conclusions

We found that the three treatment types following IGTN surgery were equally effective with regards to infection, pain, toe function and soothing effects. Our study does not prove that postoperative treatment of footbath with acidic or alkaline soap is more effective than no treatment. This is an important finding because soap treatments are time and resource intensive for patients compared with simply bandaging. Although we observed increased resistance of cutaneous skin flora to invasive pathogenic microbes following the use of acidic soaps, this did not lead to less infections than in the groups using either alkaline soap baths or bandaging. Therefore, we recommend that postoperative advice should be given on an individual basis. Furthermore, it should be taken into consideration that our study did not involve patients with high risk of infections.

Due to the limitations of our study, we recommend that larger randomised trials, also including immunocompromised patients, should be conducted to verify the findings presented here.
